# Adequate Th2-Type Response Associates with Restricted Bacterial Growth in Latent Mycobacterial Infection of Zebrafish

**DOI:** 10.1371/journal.ppat.1004190

**Published:** 2014-06-26

**Authors:** Milka Marjut Hammarén, Kaisa Ester Oksanen, Hanna Maria Nisula, Bruno Vincent Luukinen, Marko Pesu, Mika Rämet, Mataleena Parikka

**Affiliations:** 1 BioMediTech, University of Tampere, Tampere, Finland; 2 Fimlab Laboratories, Pirkanmaa Hospital District, Tampere, Finland; 3 Department of Pediatrics, Tampere University Hospital, Tampere, Finland; University of Washington, United States of America

## Abstract

Tuberculosis is still a major health problem worldwide. Currently it is not known what kind of immune responses lead to successful control and clearance of *Mycobacterium tuberculosis*. This gap in knowledge is reflected by the inability to develop sufficient diagnostic and therapeutic tools to fight tuberculosis. We have used the *Mycobacterium marinum* infection model in the adult zebrafish and taken advantage of heterogeneity of zebrafish population to dissect the characteristics of adaptive immune responses, some of which are associated with well-controlled latency or bacterial clearance while others with progressive infection. Differences in T cell responses between subpopulations were measured at the transcriptional level. It was discovered that a high total T cell level was usually associated with lower bacterial loads alongside with a T helper 2 (Th2)-type gene expression signature. At late time points, spontaneous reactivation with apparent symptoms was characterized by a low Th2/Th1 marker ratio and a substantial induction of *foxp3* reflecting the level of regulatory T cells. Characteristic *gata3/tbx21* has potential as a biomarker for the status of mycobacterial disease.

## Author Summary

Tuberculosis is a common and potentially lethal lung disease spread worldwide. One third of the world's population is estimated to be infected with *Mycobacterium tuberculosis*, yet most individuals develop a latent disease which has the potential to reactivate. Some are thought to be able to clear the infection. The current vaccine does not give adequate protection against the disease, and due to incorrect use of antibiotics, resistance to treatment has substantially increased. There is an urgent need for novel treatment approaches, such as modulation of the host's immune response. However, the ideal immune response against tuberculosis is unknown. In addition, more accurate diagnostic tools are needed for distinguishing the high risk individuals among latent patients so that treatment could be given to those that are most likely to benefit from it. In this study, we used the *Mycobacterium marinum*-zebrafish model to study the T cell responses in mycobacterial infection. Utilizing the natural heterogeneity of the zebrafish population, we found associations between the disease severity (bacterial load) and the type and magnitude of T cell responses. Our results on typical T cell signatures are useful as diagnostic biomarkers as well as provide new understanding needed for therapeutic approaches based on immunomodulation.

## Introduction

Tuberculosis (TB) is a pulmonary disease spread worldwide. It is caused by an infection with *Mycobacterium tuberculosis*. Only 5–10% of infected individuals develop a primary active disease while the most common outcome of infection is a latent or subclinical disease with no evident symptoms. This latent disease has the inherent ability to reactivate after years or even decades of latency and is therefore a major global threat. The existing vaccine, the Bacille Calmette-Guérin (BCG), is not entirely safe and does not confer protection against latent or reactivated TB. Current antibiotic regimens have started losing their efficacy due to the spread of antibiotic resistance genes [1]. In total, the primary active infections and reactivated infections cause 1–2 million deaths yearly, which makes *M. tuberculosis* the deadliest bacterium for humans [2].

The dichotomy to a latent and active tuberculosis is an over-simplification, as the infection can actually lead to a wide spectrum of disease states ranging from a well-controlled (or even cleared) latent disease to fulminant, severe forms of TB. Within the latent population, a “sub-spectrum” exists leading to differences in the risk of reactivation [3]. The pathogenesis of tuberculosis has been widely studied for decades, but as it seems that TB is not a single disease but a spectrum of different outcomes, it remains poorly understood. Better understanding on the factors that contribute to the type of TB disease is crucial for the development future treatment strategies.

The TB spectrum is likely to arise from genetic variation both in the host and in different pathogen strains as well as from environmental factors. It is known that adaptive immunity and especially T helper (Th) cells are required for controlling the disease. HIV-infected individuals are more susceptible to active and reactivated tuberculosis due to the defective T lymphocyte response [4]. Mice lacking T helper responses are hypersusceptible to TB [5]. Based on the observations that IL-12 or IFN-γ deficient mice are unable to restrict mycobacterial infection, it was initially concluded that Th1 cells are the predominant mediators of protective immunity to *M. tuberculosis* [6]–[8]. In mice, observations of an early Th1 response (2–3 weeks post infection, wpi) followed by a Th2 response simultaneously with the onset of a chronic phase, have led to a presumption that Th2 response is detrimental to the host by leading to a failure of Th1 response to clear the infection [9], [10]. Subsequently, it has also been shown that the lack of Th2 responses in IL-4, IL-13 or Stat-6 deficient mice does not lead to better resistance to *M. tuberculosis* infection but, instead, to increased bacterial burdens at later stages of infection [11]. Although the role of humoral immunity in response to mycobacterial infection is still unclear, there is evidence that Th2 responses are needed as well for optimal protective immunity [1], [12]. Despite the emerging understanding of the roles of different Th subtypes in TB immunity, it is still not known what type of Th profiles are needed at different phases of infection to provide optimal protection. In part, this is due to the lack of suitable animal models for studying the full spectrum of disease outcomes, including latency and reactivation.

Several animal models have been developed with the aim of understanding the complex pathogenesis of tuberculosis. The murine model of pulmonary TB is well standardized and has made many valuable contributions to the knowledge of the disease pathomechanisms, especially on the role of T cells as mediators of protective immunity [13]. A major constraint of the model is that mice do not develop spontaneous latency although they can restrict the bacterial growth to chronic progressive infection. One of the rare animal models developing true latency is the Cynomolgus macaque. In the macaque, a low-dose *M. tuberculosis* infection leads to active primary disease in 50% and latent disease in 50% of individuals [14]. In the rabbit model of latent TB, the lung bacterial burdens start declining at 4 wpi following a primary phase with limited bacterial growth. In the rabbit TB model, different outcomes of infection can be induced by using mycobacterial strains with different virulence properties [15], [16].

In addition to the mammalian models of TB, we have previously shown that infection of adult zebrafish with their natural pathogen, *Mycobacterium marinum*, can be used to model latent TB [17]. *M. marinum* is a close genetic relative of *M. tuberculosis*, and typically infects cold-blooded hosts, such as frogs and various freshwater and saltwater fish species [18]. *M. marinum* infection of zebrafish embryos has been established as an elegant model to dissect the innate mechanisms of protective host responses in active mycobacterial infection [19]–[21]. However, the full spectrum of mycobacterial disease outcomes can be observed only in the adult zebrafish, due to the full maturation of adaptive immune system after the first four weeks post fertilization [21], [22]. In the adult zebrafish model, the injection of a low dose of *M. marinum* (ATCC 927 type strain) into the abdominal cavity leads to a systemic infection, characterized by an initial 3–4 week phase with rapid bacterial growth, followed in most individuals by a latent phase with stable bacterial burdens. In the latently infected fish, the majority of the mycobacterial population passes into a non-replicative state, dormancy, but can be experimentally reactivated by immunosuppression [17].

The wide disease spectrum typical of mycobacterial disease results from various host- and pathogen-associated factors. It is known that genetic determinants lead to an inherent, stable preference towards either T helper 1 or T helper 2 response that varies between human individuals [23], [24]. As a starting point for our study, we hypothesized that the differences that control the T helper response might be associated with the establishment of the wide spectrum seen in TB patients and that differences in T cell polarity might be related to the progression of the disease. Taking advantage of the heterogeneity of the zebrafish population and the wide spectrum of mycobacterial disease outcomes in the zebrafish model, we set out to look for differences in T helper responses involved in regulating protective response. Finding such differences would 1) allow the use of T helper markers among latently infected individuals to distinguish between those at high or low risk of reactivation and 2) provide understanding on what type of T cell response gives the optimal protection against mycobacterial infection and allow development of novel kinds of therapeutic or preventive approaches.

## Results

### Expansion of T lymphocytes is associated with limited mycobacterial growth in the zebrafish

**Figure 1:**
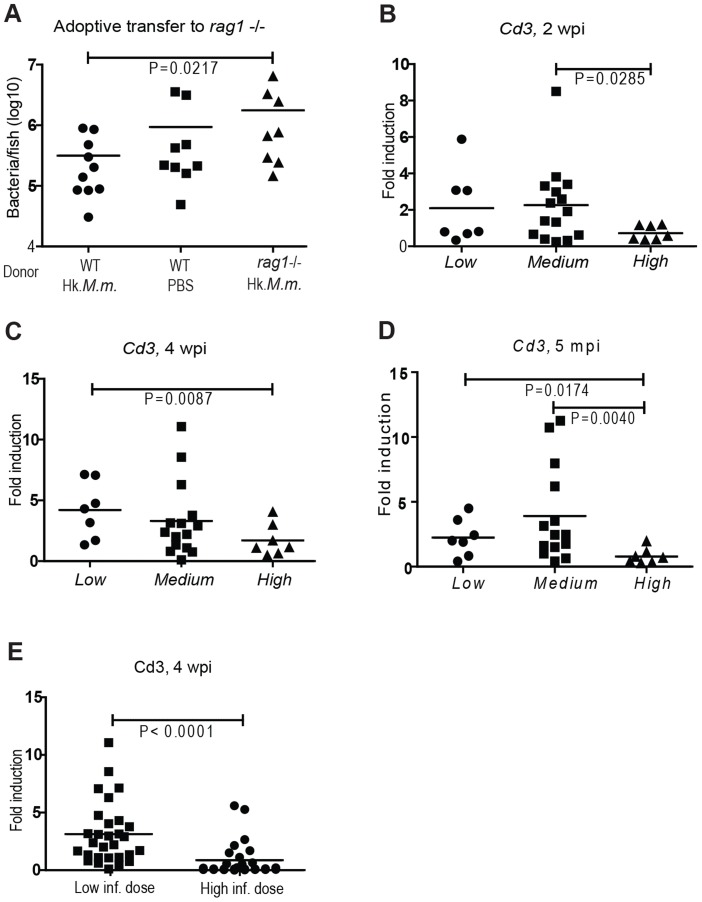
T cell numbers are higher in individuals with low bacterial loads. (A) In an adoptive transfer experiment, we transferred spleen and kidney cells to low-dose infected *rag1* (−/−) mutant fish 12 dpi. The donors were WT immunocompetent fish treated with heat-killed *M. marinum* (Hk.*M.m.*) or PBS and *rag1* (−/−) fish treated with Hk.*M.m.* 10 d prior to the adoptive transfer. The bacterial loads of the recipient fish were measured 4 wpi by q-PCR, n = 8–10/group. (B–D) At all time points of this study, zebrafish infected with a low dose of *M. marinum* (21±7 cfu) were divided in subpopulations according to the bacterial load into upper quarter (*High*) (n = 7), lower quarter (*Low*) (n = 7) and a *Medium* group (n = 15). The changes in the total T cell numbers were assessed in the different subpopulations of low-dose infected WT fish by measuring *cd3* transcription by q-RT-PCR during a primary (2 and 4 weeks) or a late stage (5 months) mycobacterial infection (E) As a control experiment for assessing the effect of initially high bacterial load on *cd3*, WT zebrafish were infected with a high dose (2691±520 cfu) and the *cd3* levels of this group (n = 25) were compared to those of the group (n = 30) infected with a low dose (21±7 cfu) at 4 wpi.

Our previous work [17] provided evidence that functional lymphocyte response is a prerequisite for latency and mycobacterial dormancy in the *M. marinum* infection of zebrafish. To further demonstrate the significance of lymphocyte responses in the immune defence against mycobacteria in zebrafish, we carried out adoptive transfer experiments on low-dose (21±7 cfu) *M. marinum*-infected *rag1* (−/−) fish. Spleen and kidney marrow cells were transferred from WT or *rag1* (−/−) zebrafish immunized with heat-killed *M. marinum* to *rag1* (−/−) recipients at 2 wpi. At 4 wpi, bacterial burdens were significantly lower in the fish that received transplants from immunized WT donors, compared to the fish that received transplants from *rag1* (−/−) donors (3.2×105 vs. 1.8×106). This indicates that heat-killed *M. marinum*-induced lymphocytes, rather than NK cells or other innate immune cells, transferred additional immune protection against *M. marinum* infection to *rag1*-deficient zebrafish. (Figure 1A).

In our current study of ∼150 individuals, a total of 10% of zebrafish were able to clear the bacterial number below the detection limit of *M. marinum* q-PCR-analysis (∼100 bacteria) (Figure S1B–D). These individuals capable of clearance were not detected in the groups that were collected at 2 wpi, suggesting that the clearance is likely to occur after the activation of adaptive responses. These results attest the significance of adaptive responses in the immune protection against zebrafish mycobacteriosis, and support the view of similarity of human and zebrafish anti-tuberculosis immunity.

In the current study, we aimed at further elucidating the details of adaptive immune response leading to a variety of infection outcomes. Unlike many other commonly used laboratory animals, zebrafish populations are genetically heterogeneous. This characteristic causes large variations and standard deviations in most studies utilizing this model, including our studies on mycobacterial infection. On the positive side, the zebrafish population provides starting material for studying the natural differences between individuals. To be able to elucidate the host factors affecting the outcome of infection, the variation in environmental and bacterial factors was first minimized: the bacterial strain, bacterial growth conditions, infection procedure, infection dose and housing of infected fish (water quality and temperature, feeding etc.) were carefully standardized.

WT adult zebrafish were infected with a low dose (21±7 cfu) of *M. marinum*, collected at various time points and divided into three subpopulations, based on their bacterial burdens. The subpopulations were named *Low* (25% of the population, individuals with the lowest bacterial burdens), *Medium* (50% of individuals) and *High* (25% of the population, individuals with the highest bacterial burdens, including the primary-progressive). The *Low, Medium* and *High* subpopulations were analyzed at various stages of mycobacterial disease: at 2 wpi (primary active disease), 4 wpi (the onset of latency in the majority of zebrafish) and 5 months post infection (mpi; late stage at which most individuals maintain latency). The reactivation risk of latent mycobacterial infection is thought to increase with increasing bacterial load [25]. The bacterial burdens in the subgroups at different time points are shown in Figure S1.

To dissect the differences in the total T lymphocyte numbers between the *Low*, *Medium* and *High* subpopulations, we quantified the cluster of differentiation (*cd*) 3 levels from internal organs of the zebrafish by q-RT-PCR. Zebrafish *cd3* has been shown to be an ortholog of the mammalian T cell marker *cd3* [26]. Here, the *cd3* expression as a marker for T cell numbers in zebrafish was further validated as described in Figure S2. Induction of T cell expansion was similar in the *Low* and *Medium* groups, seen as a 2-fold induction in *cd3* expression level already at 2 wpi (Figure 1B) and peaking to 4-fold around 4 wpi (Figure 1C), compared to the *cd3* expression levels in non-infected zebrafish. The *High* group differed from the rest of the population by showing a modest T cell expansion (max. 1.7±1.3), which was only seen at 4 wpi (Figure 1C). A similar pattern remained at a late stage of the infection (5 months, Figure 1D). To assess whether the limited T cell expansion is the cause or the consequence of enhanced bacterial growth in the *High* group, the low-dose-infected fish were compared with a group infected with a high dose of *M. marinum* (2691±520 cfu). Based on our previous work [17] a high initial dose causes the bacterial load to be significantly higher than with a low initial dose during the first 2 weeks of infection and this difference will even out by 4 wpi. At 4 wpi, *cd3* expression levels were significantly lower in the high-dose group (Figure 1E), suggesting that the reduced T cell numbers in the *High* subpopulation may at least partly be affected by the rapid bacterial growth.

Taken together, these results indicate that an early T cell expansion associates with protective response against mycobacterial infection, as the fish with highest *cd3* expression levels were always found in the *Low* and *Medium* subgroups. However, individuals with modest lymphoproliferative response were found equally in all the three subgroups, suggesting that other factors besides efficient T cell expansion are required for mounting a protective response against mycobacterial infection.

### Controlled mycobacterial infection is characterized by sufficient induction of Th2-type responses

As Th cells are potent orchestrators of immune responses during infection, it is reasonable to assume that in addition to total lymphocyte numbers, variation in Th response types may be an important factor underlying the wide spectrum of outcomes in mycobacterial infections. For zebrafish, antibody markers or reporter lines for FACS (fluorescence-activated cell sorting) analysis of different T lymphocyte populations are not available. To assess the Th1/Th2 balance of individuals with different infection outcomes, we measured the levels of master regulator transcription factors for Th1/Th2 lineage development, *T-box transcription factor 21 (tbx21)* and *gata3*, from the internal organs of infected zebrafish. Tbx21 is a Th1 cell transcription factor important for Th1 lineage commitment and gata3 is a well-known regulator of Th2 cell differentiation also playing a role in endothelial cell biology [27]. The central T cell transcription factors tbx21, gata3 and foxp3 have been identified in the fish [28]–[30]. The enrichment of *tbx21* and *gata3* in the zebrafish T cell population was validated as described in Figure S2. During infection, the alterations in the transcript levels of these transcription factors reflect the changes in the numbers of the corresponding T helper cells. The ratio of Th2/Th1 markers was used to assess the balance of T helper cell response. In addition, the induction of a Th2-type cytokine IL-4 (IL4b) [31] and a Th1-type cytokine IFN-γ (*ifnγ1-2*) was measured and the ratio was calculated.

**Figure 2:**
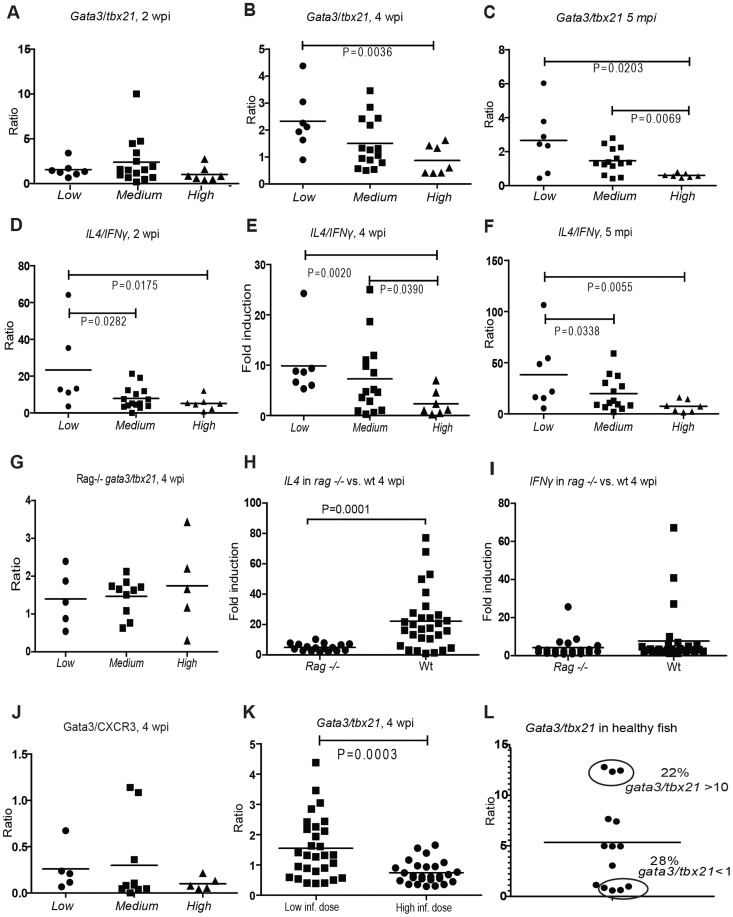
Controlled mycobacterial infection is characterized by Th2-type response from 4 weeks post infection. (A–C) *Tbx21* (*t-bet*) and *gata3* induction was measured in the different subpopulations at 2, 4 wpi and 5 mpi (months post infection). The *gata3/tbx21* ratio was calculated to determine the dominant Th type. (D–F) The induction of selected type cytokines for Th1 (*IFNγ1-2*) and Th2 response (*IL4b*) was measured in the different subpopulations. The *il4/IFNγ* ratio of induction was calculated. (G–I) *Rag1* (−/−) mutant zebrafish (n = 20) were infected with 35±18 cfu of *M. marinum* and analyzed at 4 wpi. (G) Grouping of mutant fish was carried out according to bacterial load similarly to wt fish (See Figure S1E). The association of *gata3/tbx21* with the bacterial load was assessed. (H) The induction of *il4* at 4 wpi was compared between wt and *rag1* (−/−) fish. (I) The induction of *ifnγ* at 4 wpi was compared between wt and *rag1* (−/−) fish. (J) Semi-quantitative western blots were carried out at 4 wpi from a population of 20 fish. Gata3 antibody was used as the Th2 marker, and CXCR3 as the Th1 marker. The bacterial loads were measured from the corresponding DNA samples to allow grouping to subpopulations. (K) As a control experiment for assessing the effect of initially high bacterial load on *gata3/tbx21* ratio, WT zebrafish were infected with a high dose (2691±520 cfu) and the *gata3/tbx21* ratio of this group (n = 25) was compared to those of the group (n = 30) infected with a low dose (21±7 cfu) at 4 wpi. (L) To assess the natural polarization pattern of T cells with regard to *gata3/tbx21*, WT zebrafish (n = 14) were stimulated by an intraperitoneal injection of heat-killed *M. marinum*. *Gata3/tbx21* ratio was determined 10 days post injection.

At 2 wpi, the induction of both *gata3* and *tbx21* was significantly higher in *Low* and *Medium* than in the *High* group (Figure S3A&D). At this time point, there were no significant differences in the *gata3/tbx21* ratio between the three groups (Figure 2A). At 4 wpi, *gata3* was still significantly more induced in *Low* and *Medium* groups compared to *High* group (Figure S3B). However, the *tbx21* levels were similar in all groups (Figure S3E). As determined by the *gata3/tbx21* ratio, the *Low* group had developed a significantly more Th2-biased response than the *High* group by 4 wpi (Figure 2B), suggesting that insufficiency of Th2 cells is a differentiating factor between the *Low* and *High* individuals. The *il4/ifnγ* ratios generally followed a similar pattern (Figure 2D–E). Also at 2 wpi, the *il4/ifnγ* ratio was significantly higher in the *Low* group compared the other two, although there were no significant differences in the *gata3/tbx21* ratio at this time point. At 2 weeks, it is likely that the adaptive Th response is in the process of maturation conducted by the cytokines excreted by innate immune cells. Similar patterns were observed at the late time point 5 months post infection (Figure S3C&F, Figure 2C&F).

At 4 wpi, the Th2/Th1 balance was also assessed at the protein level by semi-quantitative Western blot analysis of gata3 and CXCR3 (a CXC chemokine receptor preferentially expressed on Th1 cells) from individuals in *Low, Medium* and *High* groups (Figure 2J, Figure S4). The results showed a similar trend as seen with q-RT-PCR analyses.

To assess the importance of functional, specific lymphocytes for the changes in the levels of the markers used in this study, we also carried out similar infection experiments in *rag1* (−/−) mutants. The fish were infected with a low dose (35±18 cfu) and collected 4 wpi. We found that there was some induction of *gata3, tbx21, il4* and *ifnγ* in the infected *rag1* (−/−) mutants (Figures S2H&I and 2H&I) showing the proportion of lymphocyte-independent induction of these markers. However, the induction of *gata3*, *tbx21* and *il4* was significantly higher in the WT fish (Figures S2H&I and 2H) than in *rag1* (−/−) fish at 4 wpi. This clearly demonstrates the major contribution of functional lymphocytes in the changes seen in these markers during mycobacterial infection. The *rag1* (−/−) fish were grouped according to bacterial load (Figure S1E) as previously described for the WT fish, and association of the *gata3/tbx21* ratio and bacterial load was assessed. In the absence of functional lymphocytes no association was detected, implying that the differences in this ratio relevant to the course of mycobacterial infection seen in WT fish are indeed derived from lymphocytes. However, the expression levels of *ifnγ* were similar in *rag1* (−/−) and WT fish showing that the induction of this Th1-type cytokine in mycobacterial infection might not be as dependent on functional lymphocytes as the other markers used.

To assess whether the Th2/Th1 balance is directly influenced by the bacterial burden in the beginning of the infection, low-dose (21±7 cfu) infected fish were compared to fish infected with a high mycobacterial dose (2691±520 cfu) at 4 wpi. Average of *gata3/tbx21* ratio was found to be lower in the high-dose infected group (Figure 2K), suggesting that rapid bacterial growth can *lead to* changes in this ratio. To investigate whether the differences in the disease outcome could *result from* genetically defined Th1/Th2 preferences, we stimulated healthy WT zebrafish by an i.p. injection of heat-killed *M. marinum* and 10 days later, analyzed the *gata3* and *tbx21* transcript levels. The spectrum of individual Th2/Th1 responses was broad, similarly to that seen in humans [23]. The *gata3/tbx21* ratio varied from 0.6 to 12.8 within a group of 14 zebrafish. 22% of the individuals were substantially Th2-biased (*gata3/tbx21*>10), whereas 28% had a bias towards Th1 (*gata3/tbx21*<1) (Figure 2L). This observation of inherent Th1/Th2 phenotypes in healthy fish suggests that genetic Th1/Th2 preferences may in part lead to the development of wide disease spectrum in mycobacterial infections. Based on these results, it seems plausible that both the bacterium and the host can affect the *gata3/tbx21* ratio during mycobacterial infection.

At any of the time points of the study, the average *gata3/tbx21* ratio was never >1.0 in the progressive *High* group, whereas Th2 dominant response (average *gata3/tbx21* 1.5–2.7) was seen in the *Low* group in the primary infection. Altogether, these results show that induction of Th2-type responses during the first four weeks of mycobacterial infection are associated with controlling the bacterial growth.

**Figure 3:**
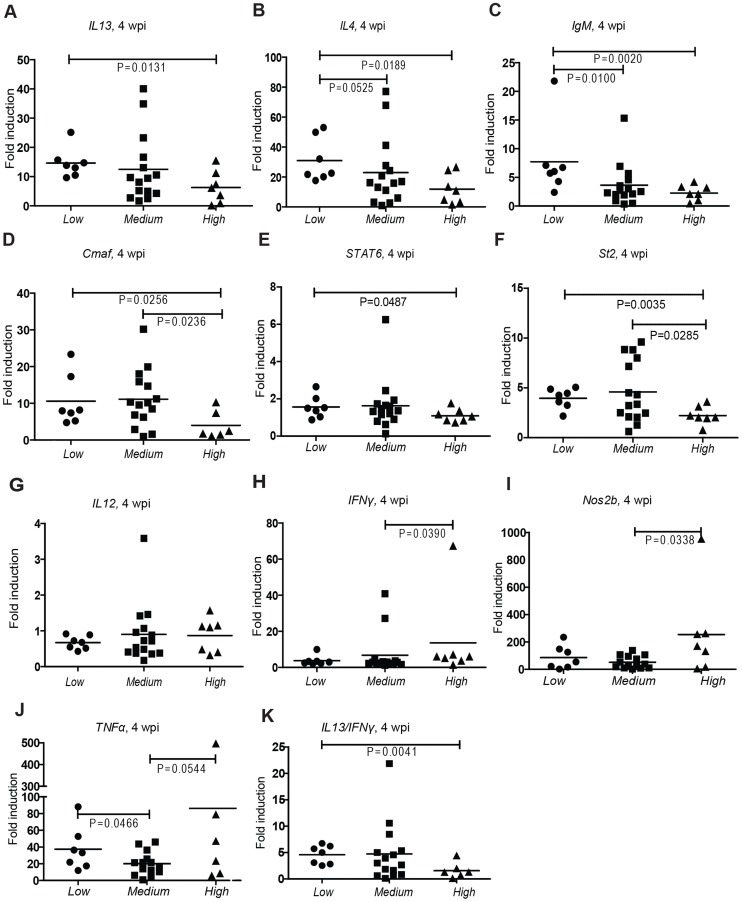
Controlled mycobacterial infection is distinguished from progressive infection by higher induction of Th2 markers. The groups with different bacterial loads were analyzed for typical Th2 (A–F) and Th1 (G–J) markers by q-RT-PCR at 4 wpi. (K) The ratio of the inductions of *il13* to *ifnγ* was also calculated in the different subgroups at 4 wpi.

In order to further confirm the reliability of our markers and to characterize the response in the different subgroups, we measured a wider selection of Th2 and Th1 signature genes at 4 wpi: Th2: interleukins *il13* and *il4, immunoglobulin M (IgM) constant region*, *V-maf musculoaponeurotic fibrosarcoma oncogene (cmaf), STAT6, St2* (Figure 3A–F) Th1: *il12, interferon gamma 1-2 (IFNγ1-2), nitric oxide synthase 2b (Nos2b), tumor necrosis factor alpha (TNFα)*;(Figure 3G–J). It has been previously shown that the expression levels of the selected Th2 marker genes are upregulated in zebrafish in response to recombinant IL-4 treatment, thus representing a Th2-type response in the zebrafish [31]. In line with *gata3* and *il4* markers, there was a significantly higher induction of Th2 signature cytokines and other Th2 related genes in the *Low* group than in the *High* group (Figure 3A–F). At the same time, Th1 markers showed a somewhat higher induction in the *High* group (Figure 3G–J). However, the differences in Th2 markers were more indicative of the bacterial burden and the disease state than those seen in Th1 markers. The *Low* group showed substantial *IgM* induction at 4 wpi differing significantly from the rest of the population (Figure 3C). Only three antibody classes exist in zebrafish, namely IgM, IgD, and fish-specific IgZ, whereas IgG, IgA and IgE are absent [32]. Secreted tetrameric IgM is the most abundant zebrafish serum immunoglobulin [32], and is induced by Th2-mediated IL-4 signaling [31]. Taken together, these data suggest that Th2-type cytokines participate in the control of bacterial growth during the first four weeks of primary mycobacterial infection.

### Th2 dominance is beneficial for the maintenance of long-term latent mycobacterial infection

**Figure 4:**
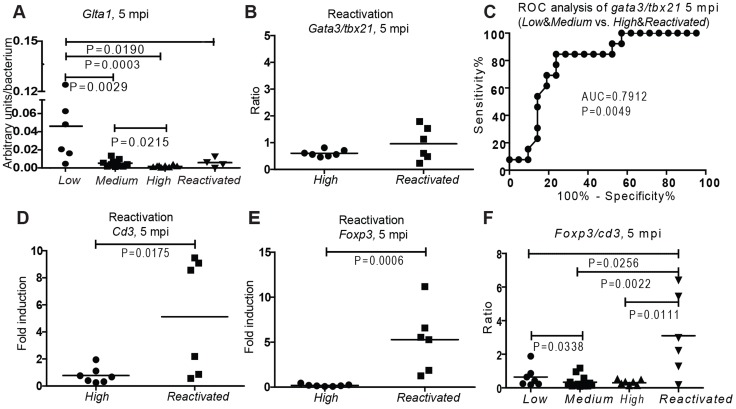
Low Th2/Th1 and high Treg are associated with activity of disease at late-stage mycobacterial infection. (A) The proportion of dormant bacteria in each non-stimulated subgroup was assessed by measuring the expression of a mycobacterial dormancy-associated gene *GltA1*. (B, D&E) The T cell inductions of *Reactivated* group (fish showing symptoms between 8 and 20 weeks after an initial controlled phase) and *High* group at 5 mpi. (C) ROC analysis of the *gata3/tbx21* ratio as a biomarker to distinguish individuals with a high bacterial burden from the individuals with a lower bacterial load (AUC = area under the curve). (F) Induction of *foxp3* normalized to *cd3* induction in the subgroups at 5 mpi and fish showing symptoms between 2 and 5 mpi.

As zebrafish with higher T cell numbers and a Th2-biased response were able to control the infection most efficiently, we next wanted to study whether these protective features were relevant for the ability of maintaining long-term latency. Even though the individuals with a primary progressive disease had already been removed from the experiment, large variations in the bacterial numbers (Figure S1D) and dormancy (Figure 4A) were seen at a late time point, when the population was again divided into subgroups according to their bacterial loads at 5 mpi. The individuals with highest levels of dormancy-associated mycobacterial citrate synthase I (*GltA1*) expression were found in the *Low* group, whereas in the *High* group *GltA1* expression levels were significantly lower than in the other groups (Figure 4A). This shows that there is a spectrum even *within* the “latent” population that has survived the primary infection.

The total T cell number still seemed to play a role at this late time point so that the *High* group had significantly less T cells than the other groups (Figure 1D). However, there was no significant difference in the total T cell number between the *Low* and *Medium* group. The protective role of Th2-type responses associated with lower bacterial numbers remained even at this late time point (Figures 2C&F). These results indicate the importance of high T cell numbers and a tendency to Th2-biased responses in determining how well the infection is controlled. It is plausible that the ideal response for inducing stable latency is similar to that required for maintaining long-term latency.

As we followed the large population of fish infected with a low initial dose, we saw that from 8 wpi to 5 mpi, there was a 17% overall morbidity in the population (data not shown). Following an eight-week period of asymptomatic infection, until the end of the 5 month follow-up, these spontaneously reactivated individuals were collected as they started showing symptoms of mycobacterial disease. These individuals had a slightly higher bacterial load than the fish in the *High* group (Figure S1D) but did not differ in terms of dormancy (Figure 4A). Thus, it is likely that the disease is active in the fish in the *High* group as well, although the fish did not yet show external signs of disease. Based on the *gata3/tbx21* ratios, the Th2/Th1 balance was similar in the *High* and *Reactivated* groups (Figure 4B), differing significantly from the *Low* and *Medium* groups. As direct assessment of the bacterial load within a population of asymptomatic humans is difficult, measuring the Th2/Th1 balance from a blood sample could be used as a clinical biomarker for estimating the activity of the disease. We carried out a ROC-analysis of *gata3/tbx21* ratio and found that *gata3/tbx21* ratio had a high sensitivity and specificity as a marker in distinguishing the high load individuals (*High*&*Reactivated*) from the well-controlling population (*Low*&*Medium*)(Figure 4C).

### Spontaneous reactivation is associated with pronounced *foxp3* induction

We next wanted to study whether there were some differences in the T cell responses between the progressive *High* and *Reactivated* groups. In the *Reactivated* group, half of the individuals showed high induction of *cd3*. This was surprising, as within all the other groups, high *cd3* was generally associated with better disease control. A possibly detrimental role of the induced T cell response in the *Reactivated* group could be explained by a *non-ideal polarization* of these cells. The most striking difference between the *High* and *Reactivated* group was the significantly higher induction of *foxp3* in the *Reactivated* group (Figure 4E), indicating a pronounced regulatory T helper (Treg) proliferation. When the expression levels of *gata3, tbx21* and *foxp3* were analyzed relative to *cd3* in the *Reactivated*, only *foxp3* was significantly different between the *Reactivated* and *High* group (P = 0.0006, 10-fold higher proportion than in the *High* group, Figure 4E). In fact, the induction of *foxp3* was higher in the *Reactivated* group compared to any of the asymptomatic groups (Figure 4F). This implies that the increase in *foxp3* expression is higher than the increase in the total T cell number and thus, the increased proportion of *foxp3* cells could be used for distinguishing *Reactivated* individuals from the rest of the population at a late stage of infection. The role of foxp3 positive cells in reactivation of tuberculosis and its applicability in diagnostics warrants further investigation.

## Discussion

It is known that T cell responses are essential in restricting mycobacterial growth in human tuberculosis as well as in various mammalian models for studying tuberculosis. The zebrafish is a newcomer in the field of immunology, and the components of its adaptive immune system have only recently been elucidated in more detail. It has been shown [33] that there are professional antigen presenting cells capable of inducing antigen-specific T cell responses in zebrafish. In our recent publication [34], we have demonstrated the presence of specific and protective immune responses against *M. marinum* infection in zebrafish. We have shown that vaccination of zebrafish with the Bacillus Calmette-Guérin (BCG) increases survival of adult zebrafish from infection with *M. marinum*. Furthermore, vaccination of zebrafish with plasmid DNA encoding mycobacterial antigens increases survival, reduces the spreading of bacteria as well as the number of granulomas in *M. marinum* infection, compared to vaccination with a control pDNA lacking the antigen-encoding sequence. *Rag1* (−/−) zebrafish lacking functional adaptive lymphocytes are not protected by the antigen-pDNA vaccine. In the antigen-pDNA -vaccinated zebrafish, interferon gamma expression levels are significantly higher during infection than in the control pDNA vaccinated fish, demonstrating the specificity of the anti-mycobacterial immune response induced in zebrafish.

For studies of zebrafish lymphocyte populations, antibody markers for FACS analysis are not available. To characterize the T cell responses of individuals with different infection outcomes, we measured expression levels of marker genes reflecting the total number of T cells and the Th profile from the internal organs of infected zebrafish. We also used the transgenic *lck:GFP* zebrafish as a tool to isolate T cells and to validate the T cell markers that were used in the study (Figure S2). We showed that the Th marker genes are enriched in sorted lck+ T cells and demonstrated a correlation between the *cd3* transcript level and the total number of lck+ cells from *lck:GFP* reporter fish. These assessments could only be done from uninfected zebrafish, because it is likely that *M. marinum* infection influences the expression of the GFP reporter gene driven by the *lck* promoter. It has been previously shown for other pathogenic mycobacteria that one of the mycobacterial virulence strategies is to inhibit host T cell receptor signaling by interfering with the expression and phosphorylation of lck [35]–[37]. Thus, the use of the *lck:GFP* reporter zebrafish line as a tool to study T cell responses in *M. marinum* infections would first require careful assessment of the effects of the bacterium on zebrafish *lck* expression. Instead, we have here relied on the use of several parallel markers reflecting the different Th profiles. In the future, development of new research tools is needed for a more detailed characterization of Th responses and their role in the pathogenesis of mycobacterial infection in the zebrafish model.

The results of the current study support the view that both Th1 and Th2 responses are induced in an optimal anti-TB-response. In the well-controlling zebrafish individuals, both Th1 and Th2-type responses were efficiently induced and showed temporal and quantitative differences compared to the Th responses of progressive individuals. The individuals that are not capable of restricting bacterial growth and, subsequently, are likely to develop a progressive disease, maintain a more Th1-biased response at all stages of infection, not reflecting an excessive Th1-type response but, instead, a lack of Th2-type induction.

Our study shows that a low-dose *M. marinum* infection elicits different types of responses in different individuals. It is known that environmental factors, such as nutritional status or infections, influence the differentiation of T helper cells. In addition to environmental factors, it has recently been demonstrated in mice and humans that each mouse strain as well as each human individual has a genetically defined Th1/Th2 bias, and that the characteristic Th phenotype is sustained over the time [23]. Generally, genetic variation in the associated transcription factors, cytokines or cytokine receptors may define the inherent individual Th bias. It is known that there are various single nucleotide polymorphisms in the enhancer regions of human Th differentiation genes, and that these polymorphisms are related to the susceptibility to various disease states [24]. In the studied heterogeneous zebrafish population, the individual outcome of mycobacterial infection can be assumed to be affected by host genetic factors, including the inherent bias in T helper phenotype, as the variation in environmental and bacterial factors is minimal. Based on the results of this study, a Th1-type response is induced equally efficiently in both progressive and well-controlling individuals, but the lack of a Th2-type response causes the disease to progress in the (genetically) susceptible population. However, in a control experiment, in which zebrafish were infected with a high initial dose of *M. marinum*, we saw that the rapid growth of bacteria may also alter the Th2/Th1 balance tilting it towards Th1. Also, we saw that the high initial dose caused the total *cd3* expression levels to remain low suggesting bacterium-induced T cell inhibition. In humans, it is known that *M. tuberculosis* can cause apoptosis of specific T cells [38] and delayed activation of CD4-positive T cells [39]. It is likely that both the bacteria and the genetic determinants of the host are capable of affecting the T cell responses in mycobacterial infection, and it is challenging to distinguish the contribution of either alone. Also, additional host factors alongside with those related T cell responses are likely to affect the disease outcome.

As latent tuberculosis exists in a major part of the human population, its spontaneous reactivation is a serious global threat. Latent tuberculosis, when not initially caused by a resistant strain, can be treated with a 9-month isoniazid monotherapy that reduces the risk of reactivation by 60–90%. However, poor treatment compliance is a common problem in treating this asymptomatic disease, as only half of the patients complete therapy [40]. The poor compliance, in turn, affects the increased antibiotic resistance to isoniazid complicating the treatment of both latent and active tuberculosis. Therefore, it would be of paramount importance to be able to recognize the small population of latently infected individuals with a higher bacterial load and to allocate the treatment to only those who are most likely to benefit from it. At a late time point, at 5 months post infection, there was a zebrafish subpopulation present with a clearly more active disease, as determined by the total bacterial load and mycobacterial dormancy gene (*GltA1*) expression. The fish with a higher bacterial load had a lower *gata3/tbx21* ratio. Based on our results, analysis of the Th1/Th2 ratio from peripheral blood mononuclear cells could provide a correlate of activity of disease among the carriers of latent *M. tuberculosis* infection. The risk of reactivation is also thought to increase with increasing bacterial loads [25], and thus the Th1/Th2 ratio could have predictive value in evaluating the risk of reactivation of a latent infection. The potential of the Th1/Th2 ratio as a biomarker in the human population warrants further investigation.

The high induction of *foxp3* expression in spontaneously reactivated individuals is in line with a previous human study showing that quantification of Foxp3 from antigen-induced peripheral blood mononuclear cells can be used to discriminate between latent and active TB [41]. During infection, regulatory T cells (Treg) have an important role in controlling excessive inflammation to prevent tissue damage, but at the same time, their immunosuppressive function can prevent bacterial clearance [42]. The role of Treg cells has been investigated during the early response to TB infection, and there is evidence that *M. tuberculosis* induces the expansion of antigen-specific Treg cells thus delaying the priming of effector T cells in the lymph nodes and the subsequent arrival of T cells to the infection site [43]. As *M. tuberculosis* is capable of such exploitation of the Treg response as part of its virulence strategy during the early TB infection, it is plausible that similar pathogen-driven expansion of antigen-specific Treg cells could also play a role in the reactivation of latent TB and the subsequent transmission of the disease. On the whole, the role of Treg cells in reactivation of latent TB is highly interesting and calls for further characterization.

The existence of individuals that are able to clear mycobacterial infection illustrates that the optimal immune response to fight TB has already developed during the evolution. Adaptive mechanisms underlying mycobacterial clearance have so far remained enigmatic, and their better understanding will undoubtedly provide valuable knowledge for drug and vaccine development against tuberculosis. The zebrafish model is uniquely suitable for dissecting the natural spectrum of mycobacterial infection in large scale population studies. Analysis of the protective immunity leading to the eradication of bacteria in zebrafish can provide valuable knowledge for the development of new innovative approaches to prevention and treatment of tuberculosis.

The importance of Th1-type response in controlling mycobacterial infection is generally recognized because mycobacteria are (facultative) intracellular pathogens. The general – and simplified – paradigm of the reciprocal regulation between Th1 and Th2 responses has led to the idea that Th2 response in tuberculosis might inhibit the bacterial clearance by Th1 immunity. Therefore most tuberculosis vaccines currently under development aim at promoting an efficient Th1 response and inhibiting the induction of a Th2 response [44]. In the studied zebrafish population, 10% of the individuals were able to clear the infection after the activation of adaptive responses (>2 wpi). These clearers had a similar, Th2-biased response as the other individuals in the well-controlling *Low* and *Medium* subgroups. On the other hand, inability to induce Th2 responses seems to be a trait that is associated with progressive mycobacterial infection in the zebrafish. Our finding argues against the paradigm of Th2 response not being useful for controlling tuberculosis. If this holds true in human TB, the current therapeutic and preventive approaches promoting Th1 and inhibiting Th2-type response need to be thoroughly reconsidered.

## Materials and Methods

### Zebrafish lines and maintenance

For most experiments, adult (5–8 month-old) wild-type AB zebrafish were used. In addition, adult, *rag1* (−/−) hu1999 mutant fish and *lck:GFP* transgenic fish (both from ZIRC) were used. Fish were kept in a flow-through system with a light/dark cycle of 14 h/10 h and were fed with SDS 400 food twice daily.

### Ethics statement

All experiments have been accepted by the Animal Experiment Board in Finland (under the Regional State Administrative Agency for Southern Finland) and were carried out in accordance with the EU-directive 2010/63/EU on the protection of animals used for scientific purposes and with the Finnish Act on Animal Experimentation (62/2006). Permit for the zebrafish facility: LSLH-2007-7254/Ym-23, Permit for experiments: ESAVI/6407/04.10.03/2012, PH1267A and ESAVI/733/04.10.07/2013.

### Experimental infection

*M. marinum* (ATCC 927) was cultured as described in [17]. In brief, bacteria were grown at 29°C in standard mycobacterium medium 7H9 (BD) with standard additives to an OD600 of 0.495–0.680 Anesthetized fish were intraperitoneally (i.p.) injected with 5 µl of bacteria suspended in sterile PBS using an Omnican 100 30 G insulin needle (Braun, Melsungen, Germany). The bacterial dose was verified by plating on 7H10 (BD) with the standard additives. The low infection dose was 21±7 cfu and the high dose 1783±364 cfu.

### Injections with heat-killed *M. marinum*

*M. marinum* (ATCC 927) was transferred from 7H10 plate into 10 ml of liquid 7H9 medium with standard additives and cultured for 3–4 days at 29°C to an OD600 of 0.490. Pelleted bacteria were resuspended in PBS corresponding to half of the original culture volume. The bacteria were heat-killed at 100°C for 20 min and thereafter homogenized for 4 min with 4000 rpm using homogenization tubes from Mobio (California, USA) and Mobio PowerLyzer24 bead beater. Samples were plated on 7H10 and LB to verify proper killing. Heat-killed bacteria were injected in a volume of 5 µl *i.p* using Omnican 100 30 G insulin needles (Braun).

### Transplantation of lymphatic cells from AB to *rag1* (−/−) fish

Kidney and spleen were collected from a euthanized AB fish in 20 µl of sterile PBS. The organs were gently homogenized by pipetting up and down ∼20 times. 10 µl of this suspension was injected *i.p.* into an anesthetized recipient *rag1* (−/−) fish.

### FACS sorting

For lymphocyte sorting experiments, *lck:GFP* fish were euthanized and their internal organs collected in ice-cold HBSS supplemented with 2% FBS (both from Life technologies, CA, USA). The tissue was mechanically disrupted by pipetting and passed through a 50 µm cell strainer to prepare single cell suspensions. Cells were washed twice with cold HBSS (+FBS), pelleted at 4°C, 300 g for 5 min and resuspended in 1 ml of the same buffer. 1 ml of Histopaque-1077 (Sigma-Aldrich, MO, USA) was then added under the cell suspension and lymphocytes and other mononuclear cells were enriched by centrifugation at room temperature for 20 min, 400 g. After centrifugation, the middle phase containing the target cells was transferred into a new tube, washed once and resuspended in HBSS (+FBS). Lck+ lymphocytes were sorted with FACSAria I (BD) (purity ≥95% based on GFP expression), collected by centrifugation and RNA was extracted using TRI reagent as described in [17].

### q-PCR

The samples for gene expression analysis and mycobacterial quantitation were prepared using TRI reagent for DNA-RNA co-extraction (MRC, OH, USA) as previously described in [17]. RNA samples were treated with DNAse (Fermentas) according to the manufacturer's protocol. Bacterial loads were measured by q-PCR from DNA samples using SENSIFAST NO-ROX SYBR kit with *M. marinum*-specific primers as described in [17]. A dilution series of DNA extracted from mycobacterial culture was included in each run to allow absolute quantification. Gene expression was measured by q-RT-PCR using Bio-Rad iScript One-Step RT-PCR Kit with SYBR Green with various primers. Host genes were normalized to *glyceraldehyde 3-phosphate dehydrogenase* (*GAPDH*) or to *elongation factor 1 alpha (Ef1a)*, and the mycobacterial dormancy gene *GltA1* was normalized to the total bacterial load. q-RT-PCR results were analyzed using the ΔCt method. The induction of host genes was compared to a baseline RNA sample extracted from a pool of healthy, non-infected zebrafish and shown as fold induction compared to average normal levels. *GltA1* levels are shown in arbitrary units. Gene accession numbers and primer sequences can be found in Text S1.

### Protein extraction and western blotting

For Western blotting, fish were collected 4 weeks after infection with 21±7 cfu. The peritoneal cavity of the euthanized fish was emptied and the collected organs were homogenized in 1.5 ml of TRI reagent (MRC, OH, USA) using the PowerLyzer24 bead beater. RNA-DNA co-extraction was carried out as described in [17]. After DNA extraction, the remaining interphase and organic phase were used for protein extraction according to the TRI reagent manufacturer's protocol. In brief, proteins were precipitated by adding 3 volumes of acetone and pelleted at 12,000 g for 10 min at 4°C. The protein pellet was washed three times with 0.8 ml of 0.3 M guanidine hydrochloride in 95% ethanol supplemented with 2.5% glycerol (v:v) and once with 1 ml of ethanol containing 2.5% glycerol (v:v). For solubilization of the protein pellet, 0.1 ml of 1% SDS per 10 mg of tissue sample was used. For Western blotting, 40 µg of total protein was resolved on a 10% SDS-PAGE gel and blotted onto Amersham Hybond ECL nitrocellulose membrane (GE Healthcare, Little Chalfont, UK). The following primary antibodies were used: anti-Gata-3 (IN) Z-Fish (AnaSpec, California, USA), anti-CXCR-3.2 (IN) Z-Fish (AnaSpec), anti-GFP antibody NB600-303 (Novus biological, Colorado, USA). In addition, actin was detected from all the membranes with anti-actin (MAB1501) antibody (Millipore, Temecula, USA) for sample normalization. IRDye infrared secondary antibodies (LI-COR Biosciences, Nebraska, USA) and Odyssey CLx (LI-COR) were used for target protein detection and Image Studio software (LI-COR) was used for protein quantitation. A representative image of the blots showing 10 individuals can be found in the Supplementary material (Figure S4).

### Statistical analysis

Statistical analysis was carried out using the GraphPad Prism software (5.02). For determination of statistical significance of differences between the different groups, a non-parametric one-tailed Mann-Whitney test was used, if not stated otherwise. P-values<0.05 were considered significant. For estimating the predictive value of *gata3/tbx21* and *foxp3* expression for activity of the disease a ROC analysis was carried out with a confidence interval of 95%. AUC (area under curve) value of 0.5 indicates no connection and 1.0 indicates a perfect marker.

## Supporting Information

Figure S1 **Bacterial loads in the different subgroups at different stages of the infection.** Organs from infected WT fish were collected at various time points (A) 2 wpi, (B) 4 wpi, (C) 7 wpi and (D) 5 mpi. The bacterial loads were measured by q-PCR. Based on the bacterial load, the fish were grouped in upper and lower quartile (*High* and *Low*, respectively) and the middle 50% (*Medium*). (D) Between 2 and 5 months post infection, fish showing external signs of disease were euthanized and labeled the *Reactivated* group. The bacterial loads of the *Reactivated* fish are shown with the bacterial loads from the fish collected at 5 mpi. (E) Organs were collected from low-dose *M. marinum*-infected *rag1* (−/−) fish at 4 wpi. The bacterial loads were measured by q-PCR and the fish were grouped as described for WT fish above.
(TIF)

Figure S2 **Validation of markers by FACS-enrichment of T cells using** ***lck:GFP*** **reporter line.** (A) The internal organs of non-infected *lck:GFP* reporter fish were collected and mononuclear cells (including lymphocytes) were enriched by Histopaque-1077 gradient centrifugation. The cells were then sorted based on size, granularity and GFP expression. (B–F) The marker gene expression was measured from sorted T cell samples by and compared to that measured from an unsorted tissue block. (G) The linear correlation between the T cell count of the sample and *cd3* expression measured by q-RT-PCR was assessed; R2 = 0.81. (H–I) *Rag1* (−/−) mutants and WT zebrafish were infected with a low dose of *M. marinum* and analyzed for *Tbx21* and *Gata3* expression by q-RT-PCR at 4 wpi.
(TIF)

Figure S3 **The induction levels of** ***gata3*** **and** ***tbx21*** **.** The induction levels of *gata3* (A–C) and *tbx21* (D–F) are shown separately in the different subgroups at 2 wpi (A&D), 4 wpi (B&D) and 5 mpi (C&F).
(TIF)

Figure S4 **Semi-quantitative western blots on a Th1 and Th2 markers are in line with the results gained from q-PCR data.** Western blots were carried out at 4 wpi from a population of 20 fish. Here shown as a representative the blots of 10 individuals (numbered 1–10). Th2/Th1 ratio was assessed with anti-Gata-3 (IN) and anti-CXCR-3.2 (IN) antibodies. Actin was detected for sample normalization.
(TIF)

Text S1 **Primer sequences and the accession numbers of target genes.**
(DOC)
