# Supplementary figures and images for: Adequate Th2-Type Response Associates with Restricted Bacterial Growth in Latent Mycobacterial Infection of Zebrafish
Source: PLoS Pathog. 2014 Jun 26;10(6):e1004190. doi: 10.1371/journal.ppat.1004190 (PMC4072801; doi:10.1371/journal.ppat.1004190)

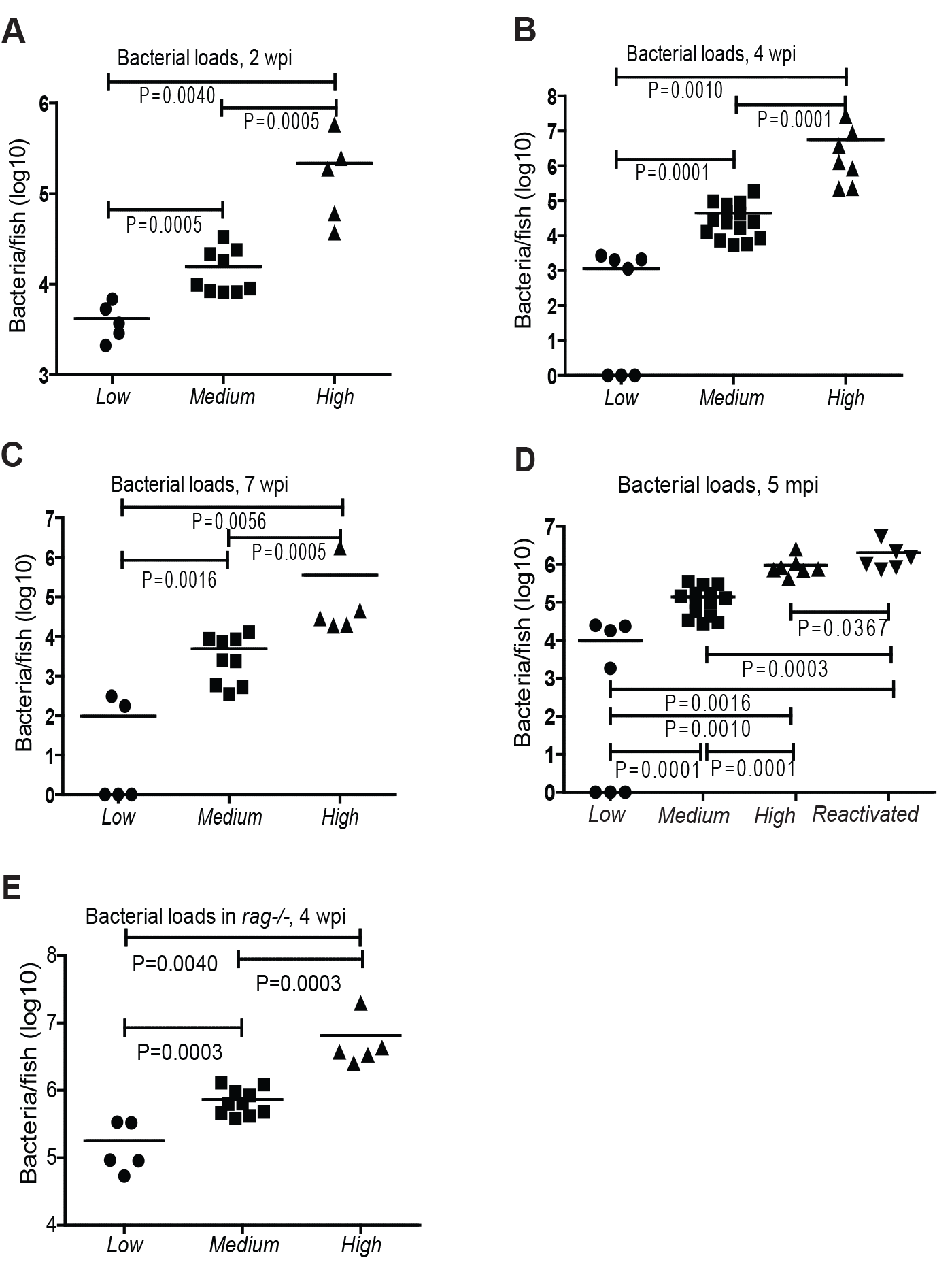

Supplement: Figure S1 — Bacterial loads in the different subgroups at different stages of the infection. Organs from infected WT fish were collected at various time points (A) 2 wpi, (B) 4 wpi, (C) 7 wpi and (D) 5 mpi. The bacterial loads were measured by q-PCR. Based on the bacterial load, the fish were grouped in upper and lower quartile (High and Low, respectively) and the middle 50% (Medium). (D) Between 2 and 5 months post infection, fish showing external signs of disease were euthanized and labeled the Reactivated group. The bacterial loads of the Reactivated fish are shown with the bacterial loads from the fish collected at 5 mpi. (E) Organs were collected from low-dose M. marinum-infected rag1 (−/−) fish at 4 wpi. The bacterial loads were measured by q-PCR and the fish were grouped as described for WT fish above. (TIF) [file ppat.1004190.s001.tif]

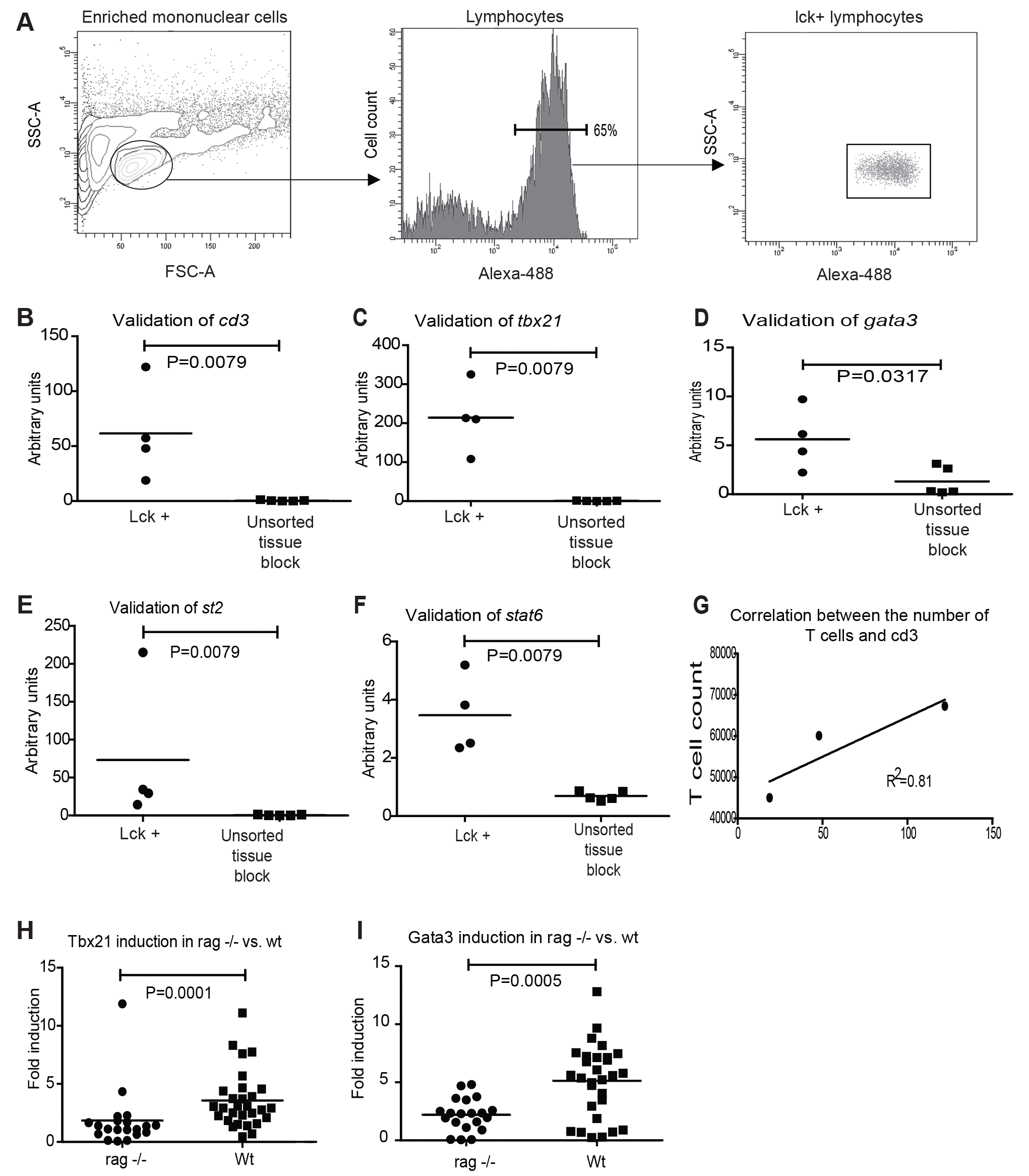

Supplement: Figure S2 — Validation of markers by FACS-enrichment of T cells using lck:GFP reporter line. (A) The internal organs of non-infected lck:GFP reporter fish were collected and mononuclear cells (including lymphocytes) were enriched by Histopaque-1077 gradient centrifugation. The cells were then sorted based on size, granularity and GFP expression. (B–F) The marker gene expression was measured from sorted T cell samples by and compared to that measured from an unsorted tissue block. (G) The linear correlation between the T cell count of the sample and cd3 expression measured by q-RT-PCR was assessed; R2 = 0.81. (H–I) Rag1 (−/−) mutants and WT zebrafish were infected with a low dose of M. marinum and analyzed for Tbx21 and Gata3 expression by q-RT-PCR at 4 wpi. (TIF) [file ppat.1004190.s002.tif]

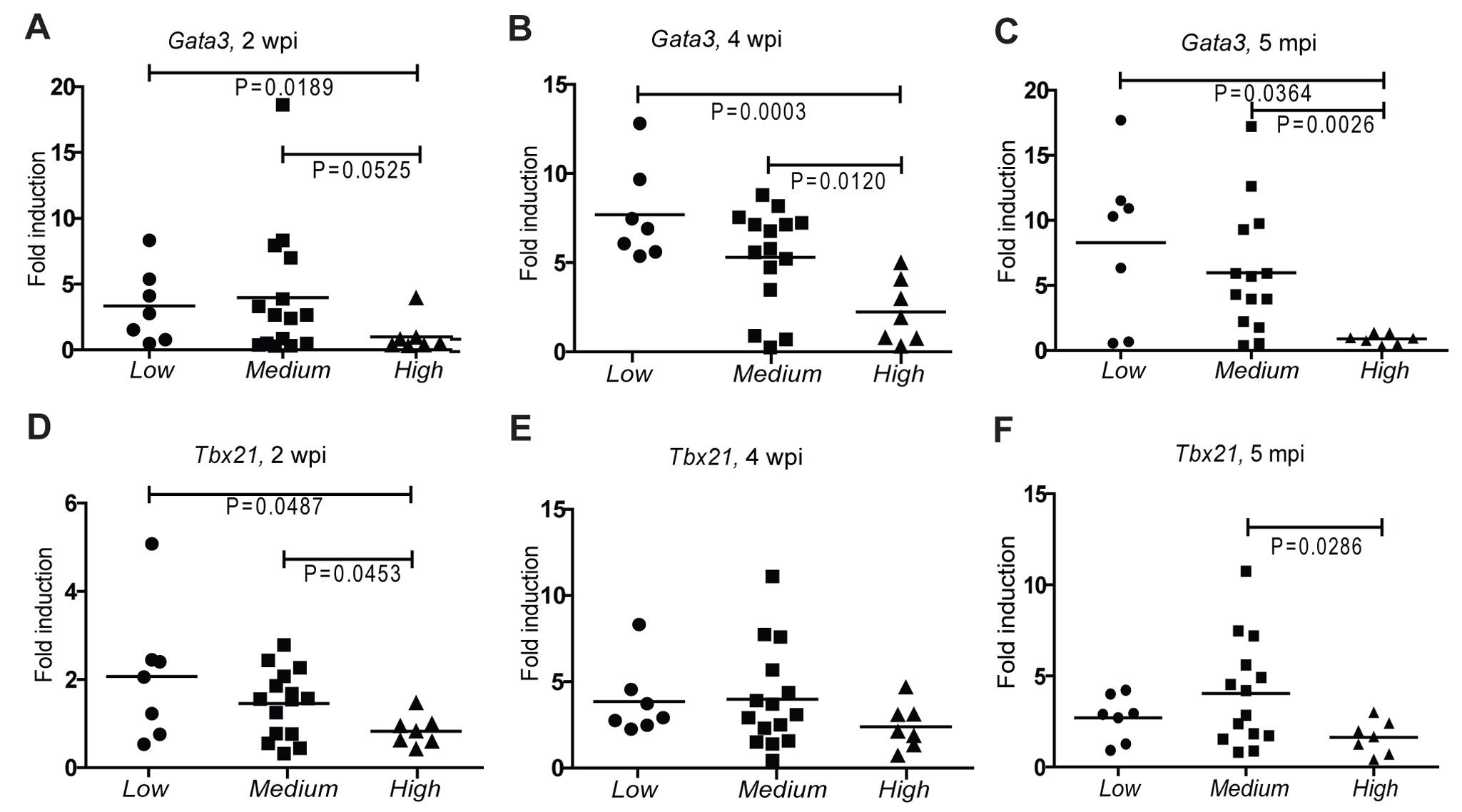

Supplement: Figure S3 — The induction levels of gata3 and tbx21 . The induction levels of gata3 (A–C) and tbx21 (D–F) are shown separately in the different subgroups at 2 wpi (A&D), 4 wpi (B&D) and 5 mpi (C&F). (TIF) [file ppat.1004190.s003.tif]

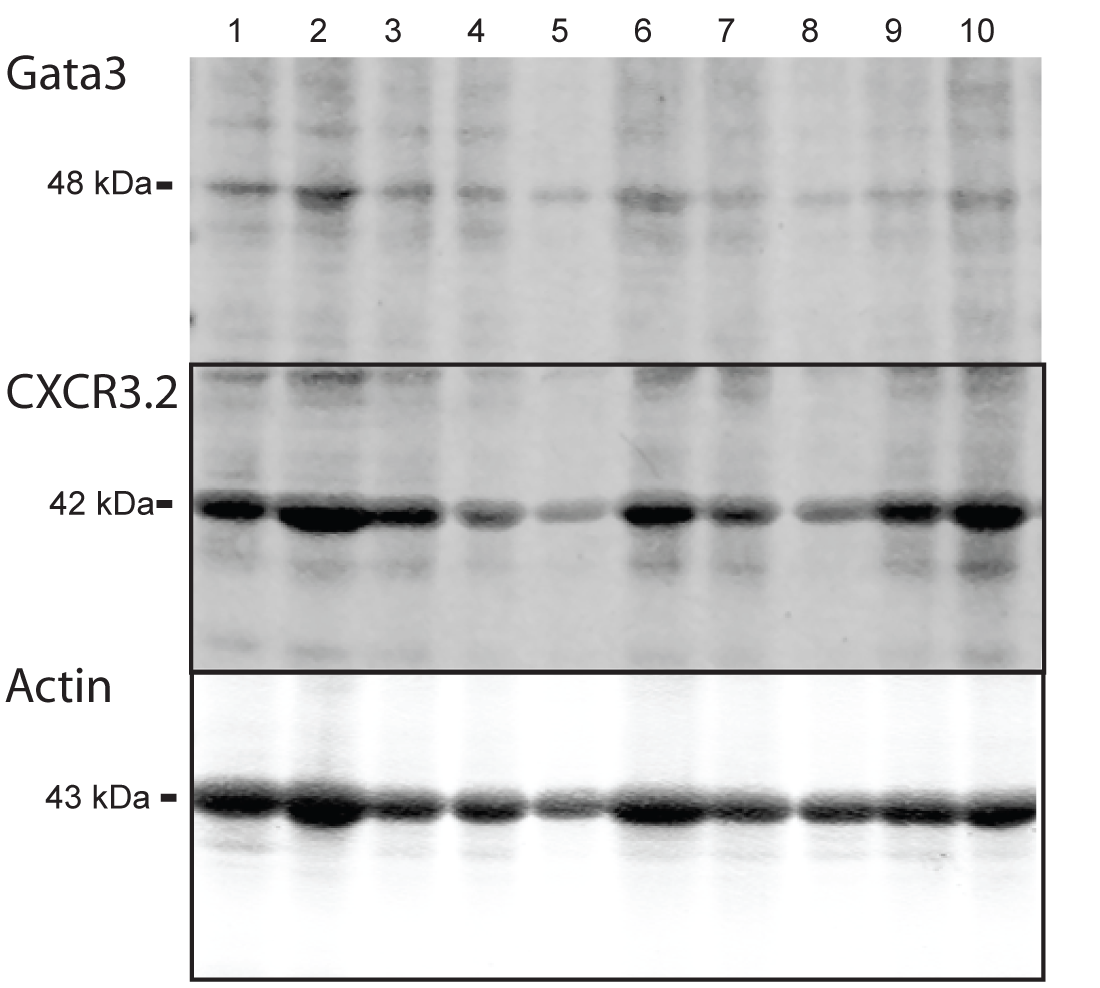

Supplement: Figure S4 — Semi-quantitative western blots on a Th1 and Th2 markers are in line with the results gained from q-PCR data. Western blots were carried out at 4 wpi from a population of 20 fish. Here shown as a representative the blots of 10 individuals (numbered 1–10). Th2/Th1 ratio was assessed with anti-Gata-3 (IN) and anti-CXCR-3.2 (IN) antibodies. Actin was detected for sample normalization. (TIF) [file ppat.1004190.s004.tif]
