# Supplementary material for: Adequate Th2-Type Response Associates with Restricted Bacterial Growth in Latent Mycobacterial Infection of Zebrafish
Source: PLoS Pathog. 2014 Jun 26;10(6):e1004190. doi: 10.1371/journal.ppat.1004190 (PMC4072801; doi:10.1371/journal.ppat.1004190)
Supplement: Text S1 — Primer sequences and the accession numbers of target genes. (DOC) [file ppat.1004190.s005.doc]

**Primers and accession numbers of target genes**

**Zebrafish (*Danio* rerio)**

*Tbx21* ZDB-GENE-080104-3 F: GGCCTACCAGAATGCAGACA, R: GGTGCGTACAGCGTGTCATA

*Gata3* ZDB-GENE-990415-82 F: GGATGGCACCGGTCACTATT, R: CAGCAGACAGCCTCCGTTT

*IL4* ZDB-GENE-100204-1 F: GCAGGAATGGCTTTGAAGGG R: GCAGTTTCCAGTCCCGGTAT

*IL13* ZDB-GENE-100727-2 F: GGAAGCTGTGTTAGTCAATCC R: GCCTGACAGAAATAATCATGC

*FOXP3a*  ZDB-GENE-061116-2 F: CAAAAGCAGAGTGCCAGTGG, R: CGCATAAGCACCGATTCTGC

*IL12* ZDB-GENE-060724-1,F:AGCATGGCTCTGGCTCTGGC, R:TGCTCCTTCATCTTTCCCTCCTTCT

*IFNγ1-2* ZDB-GENE-040629-1 F: GGGCGATCAAGGAAAACGACCC, R: TAGCCTGCCGTCTCTTGCGT

*Nos2b* ZDB-GENE-080916-1F: TCACCACAAAAGAGCTGGAATTCGG, R:ACGCGCATCAAACAACTGCAAA

*TNFα* ZDB-GENE-050317-1 F: GGGCAATCAACAAGATGGAAG, R: GCAGCTGATGTGCAAAGACAC

*GAPDH* ZDB-GENE-030115-1 F: AGTGTCAGGACGAACAGAGGCT, R:GCCAATGCGACCGAATCCGTTA

*EF1α* ZDB-GENE-990415-52 F: CTGGAGGCCAGCTCAAACAT, R: ATCAAGAAGAGTAGTACCGCTAGCATTAC

*ST2* ZDB-GENE-060621-4 F: CGCTTTACTGTGGAGAGATGG, R: TGCTGCTGTGTTTGATGCTC

*Stat6* ZDB-GENE-030131-9359 F: GGTAGTCAGGAAATCAATGC, R: ACCTCAGACATGAACTTACTGC

***Mycobacterium marinum***

*Glta1* MMAR_1381 F: CCACAGCCACATGAGTTACG, R: GCTCGAAGGTATCCACAACC

16S–23S ITS, locus AB548718 (for bacterial quantification): F:caccacgagaaacactccaa, R: acatcccgaaaccaacagag
